# Prognostic and immunological significance of calcium-related gene signatures in renal clear cell carcinoma

**DOI:** 10.3389/fphar.2022.1055841

**Published:** 2022-12-14

**Authors:** An Liu, Fei Li, Bao Wang, Le Yang, Hai Xing, Chang Su, Li Gao, Minggao Zhao, Lanxin Luo

**Affiliations:** ^1^ Precision Pharmacy and Drug Development Center, Department of Pharmacy, Tangdu Hospital, Fourth Military Medical University, Xi’an, Shaanxi, China; ^2^ Department of Pharmacy, The Hospital of 92880 Troops, PLA Navy, Zhoushan, Zhejiang, China; ^3^ Department of Neurosurgery, Tangdu Hospital, Fourth Military Medical University, Xi’an, Shaanxi, China; ^4^ Medical Affairs Division, Eastern Hepatobiliary Surgery Hospital, Second Military Medical University, Shanghai, China; ^5^ Shaanxi Provincial Corps, Chinese People’s Armed Police Force, Xi’an, Shaanxi, China; ^6^ Institute of Medical Research, Northwestern Polytechnical University, Xi’an, Shaanxi, China

**Keywords:** calcium, kidney renal clear cell carcinoma, prognosis, immunity, immune response

## Abstract

**Background:** Calcium signaling is implicated in multiple processes including immune response that important in tumor progression. Kidney renal clear cell carcinoma (KIRC) is the most frequent histological type of renal cell carcinoma with up to a third of cases develop metastases. As a result of a lack of in-depth understanding of the mechanisms underlying KIRC, treatment options have been limited. Here, we aim to comprehensively investigate the landscape of Ca^2+^ channels, pumps and exchangers in KIRC patients.

**Methods:** The mRNA expression profiles and gene variations of 58 calcium-related genes (CRGs) in KIRC patients and normal control cases were downloaded from TCGA database. CRGs-related risk score was constructed to quantify calcium patterns by using least absolute shrinkage and selection operator (LASSO) regression. The prognostic value, biological functions, immune landscape and therapeutic sensitivities based on CRGs-related risk score were then evaluated using multiple methods. Finally, key gene of CRGs was identified by weighted gene co-expression network analysis (WGCNA). TCGA-CPTAC, GSE53757 datasets, as well as human tissues were used for validation.

**Results:** KIRC patients had significant differences in CRG expression, prognosis, and biological functions between two CRG clusters. CRGs-related risk score was then determined. The prognosis, tumor mutation burden, immune cell infiltration, immune checkpoints, and the response of targeted inhibitors were remarkably different between high and low CRGs-related risk subtypes. CRGs-related high-risk subtype was characterized by immunosuppressive microenvironment with poor prognosis. Meanwhile, several targeted drugs showed distinct sensitivity between CRGs-related risk subtypes. Finally, TRPM3 was identified as a key CRG based on risk score in KIRC patients. TRPM3 mRNA and protein expression were significantly lower in KIRC tumors than in normal controls. Low TRPM3 expression was associated with poor prognosis in KIRC patients.

**Conclusion:** Our study highlighted the promising prognostic value of CRGs in KIRC tumors. The evaluation of CRGs-related risk score will contribute to predicting prognosis and clinical therapy in KIRC patients.

## 1 Introduction

Renal cell carcinoma represents a group of histologically and molecularly heterogeneous tumors that consists of three major subtypes, including kidney renal clear cell carcinoma (KIRC), kidney renal papillary cell carcinoma (KIRP) and kidney chromophobe (KICH) (Inamura, 2017; Wm and Cj, 2019). The incidence of renal cell carcinoma is increasing annually with approximately 400,000 new cases diagnosed worldwide per year (Petejova and Martinek, 2016; Bray et al., 2018; Klatte et al., 2018). In the year of 2018, 403,262 new cancer cases (9.1% of the total cases) and 175,098 cancer deaths (3.7% of the total cancer deaths) occurred globally (Chen et al., 2020). Among renal cell carcinoma subtypes, KIRC is the most frequent histological type with a prevalence of 70% individuals diagnosed (Bray et al., 2018). The disease can be caught early and effectively treated with surgery, but it is almost always fatal if metastases develop (Jonasch et al., 2014; Bray et al., 2018). It is estimated that patients with metastatic KIRC will survive on average 13 months and that the 5-year survival rate will not exceed 10% (Petejova and Martinek, 2016). Treatments for KIRC are limited owing to the resistance to chemo-/radio-therapies and a lack of in-depth understanding of the underlying mechanisms (Petejova and Martinek, 2016; Ren et al., 2022). As a result, it is an urgent need to explore novel biomarkers that can help improve precision treatments of KIRC patients.

Intracellular calcium ion (Ca^2+^), as a second messenger, plays direct and robust roles in many biological processes (Prakriya and Lewis, 2015). Nowadays, increasing evidence show calcium signaling is also responsible for tumorigenesis and progression (Zhong et al., 2019; Wu et al., 2021). It is clearly that Ca^2+^ act as novel and important regulator in specific processes of tumor development, covering all major hallmarks of cancer progression (Ibrahim et al., 2019). These processes are mediated by calcium channels, pumps, and exchangers. The associations between calcium signaling and renal cell carcinoma have been little recognized. Earlier studies found decreased expression of TRPV5 and TRPV6 in human renal cell carcinoma (Wu et al., 2011). The absence of TRPC4 in renal cell carcinoma results in impaired Ca^2+^ intake, misfolding, retrograde transport, and diminished antiangiogenic thrombopsonin-1 production, thus enabling angiogenic switch during tumor progression (Veliceasa et al., 2007). Orai1 and STIM1 are essential for tumor cell migration and proliferation of KIRC (Kim et al., 2014). Recent study also revealed an increased risk of renal cell carcinoma in users of dihydropyridine calcium channel blockers (Kristensen et al., 2020). TRPM7 has been reported to regulate migration and invasion of KIRC tumor cells *via* phosphorylating Akt signaling pathways (Ha et al., 2018; Zhao et al., 2018). However, little is known about the gene signatures of calcium channels, pumps, and exchangers, in KICH prognosis and biological functions, which needs further and comprehensive investigation.

In this study, we integrated calcium-related genes (CRGs) and identified them in prognosis, biological functions, immune landscape, and the response of clinical therapeutics in KIRC patients in the public databases. As a surprise, we found that different risk subtypes associated with CRGs had different prognoses and unique immunological characteristics. The data highlighted the promising prognostic role of CRGs in KIRC tumors.

## 2 Materials and methods

### 2.1 Data collecting

A comprehensive set of clinical datasets with detailed clinical annotations was gathered from the Cancer Genome Atlas (TCGA) and GEO database (GSE53757) which were further processed *via* TCGAbiolinks and manually. A total of 541 KIRC patients with 72 normal cases from TCGA-KIRC, 220 KIRC patients with 149 normal cases from TCGA-CPTAC datasets, and 72 KIRC patients with paired normal tissues from GSE53757 were included in the study. Data were displayed as transcripts per kilobase million (TPM). The sample information from these three datasets has been included in Supplementary Tables S1–S3.

### 2.2 Patient tissue collecting

KIRC tissue microarray, including 70 tumor specimens and their matched adjacent normal tissues from stage I to stage III, was obtained from Shanghai Zhuoli biotechnology Co., Ltd. (Shanghai, China). In addition, six paired tumor-normal tissues were collected at Eastern Hepatobiliary Surgery Hospital, Second Military Medical University following approval by the institutional review boards. The sample information has been included in Supplementary Tables S4, S5.

### 2.3 Somatic mutations and copy number variations of 58 calcium-related genes (CRGs)

To determine the mutational burden of 58 CRGs, we counted the total number of non-synonymous mutations from TCGA cohort. The somatic mutations driver genes were evaluated using “maftool” R package. Further investigation of the top driver genes was conducted using UCSC Xena datasets, which contained information on copy number variations (CNVs) (https://xenabrowser.net/datapages/) (Goldman et al., 2020).

### 2.4 Unsupervised clustering based on 58 CRGs

The intracellular Ca^2+^ concentration is regulated by calcium channels, pumps, and exchangers. Regarding the channels, there are three main types, including voltage-gated calcium channels (VGCCs), transient receptor potential (TRP) channels, and calcium release-activated Ca^2+^ (CRAC) channels (Wu et al., 2021). Ca^2+^-ATPase pumps and Na^+^/Ca^2+^ exchangers (NCX) also play important roles in calcium signaling. In this study, we selected and summarized 58 CRGs that belong to the above categories (Supplementary Table S6). Unsupervised clustering of CRG expression from these datasets was carried out using the R package “ConsensusClusterPlus” *via* k-means clustering algorithm with Euclidean distance (Wan et al., 2021; Zhang et al., 2022).

### 2.5 Biological functions based on CRG clusters

Based on expression profiling, gene set variation analysis (GSVA) estimates the variation of gene set enrichment in a non-parametric, unsupervised manner. By calculating each sample’s GSVA score, the R package “GSVA” was used to quantify HALLMARK, KEGG, and GO-related pathway activity (Hänzelmann et al., 2013; Li et al., 2022).

### 2.6 CRGs-related risk score construction

A Cox proportional hazards regression analysis was performed on each CRG in TCGA cohort to screen significant genes that associated with survival (Nagashima and Sato, 2017). Those genes were then regressed using LASSO Cox (Tibshirani, 1997). The detailed information has been described previously (Yin et al., 2021). CRGs were modeled with a multivariate model. The risk score was calculated based on genes with non-zero coefficients.

### 2.7 Immune landscape depiction between CRGs-related risk subtypes

As a method of assessing and quantifying immune cell infiltration in each sample, single-sample gene-set enrichment analysis (ssGSEA) by R packages “GSVA” and “GSEABase” was employed (Yin et al., 2021). Based on ssGSEA enrichment score, immune cell infiltration abundance was calculated for each cell type. As a final analysis, immune cell infiltration differences between CRG-related risk subtypes were examined.

An immune checkpoint assay was performed on a single sample, which contained 27 molecules from the B7-CD28 superfamily, the TNF superfamily and other molecules. The co-stimulatory and co-inhibitory checkpoints were also applied to explore the differences in CRGs-related risk subtypes.

### 2.8 Therapeutic sensitivity prediction between CRGs-related risk subtypes

The difference in therapeutic responses between CRGs-related risk subtypes from targeted therapies was analyzed. With the help of the R package “oncoPredict,” we calculated the concentration that causes 50% growth reduction (IC50) (Maeser et al., 2021). A Wilcoxon rank-sum test was used to compare differences in IC50 for CRGs related risk subtypes.

### 2.9 Key gene selection

WGCNA was applied to identify key prognostic CRG based on the intersection of prognostic genes and risk scores as described previously (Zhang et al., 2022). Briefly, a suitable power exponent was selected to convert the adjacency matrix to the topological overlap matrix. Following that, a correlation analysis was conducted between the gene consensus modules and the risk score. The modules negatively and positively correlated with risk score were selected for subsequent analyses. Genes identified by the intersection of the module genes and the CRGs were identified as key gene.

### 2.10 Immunohistochemistry (IHC)

The protein level of TRPM3 was detected by IHC. In brief, after routine deparaffinization, rehydration, blocking and antigen retrieval, the slides were incubated overnight at 4°C with primary TRPM3 antibody (#DF14592, Affinity Biosciences, Suzhou, China) followed by incubation with HRP-conjugated secondary antibody and visualized with the EnVision Detection Kit (DAKO). The nucleus was stained with hematoxylin. The immunostaining score was calculated by the Visiopharm software.

### 2.11 RNA extraction and RT-PCR

An extraction of total RNA was performed with Trizol (Sangon Biotech, Shanghai, China). As part of the synthesis process, the cDNA was synthesized using the Transcriptor First Strand cDNA Synthesis Kit (Roche, Mannheim, Germany). The primer sequence of TRPM3 and β-actin were as follows: TRPM3, 5′-CTC​CAG​TTT​TTC​CCC​TTT​CC-3′ (forward), and 5′-CCC​TTC​CTT​CTC​CCT​CTC​TT-3′ (reverse); β-actin, 5′-GAA​GAG​CTA​CGA​GCT​GCC​TGA-3′ (forward), and 5′- CAG​ACA​GCA​CTG​TGT​TGG​CG-3′ (reverse).

### 2.11 Statistical analysis

R project (version 4.1.2, http://www.r-project.org/) was used for all statistical analyses. Based on CRG clusters or CRG-related risk subtypes, Kaplan-Meier analysis was used to evaluate the survival time of KIRC patients. In the study, Spearman correlation was used to assess correlations. Student’s *t*-test and one-way ANOVA were used to evaluate differences between groups for variables.

## 3 Results

**FIGURE 1:**
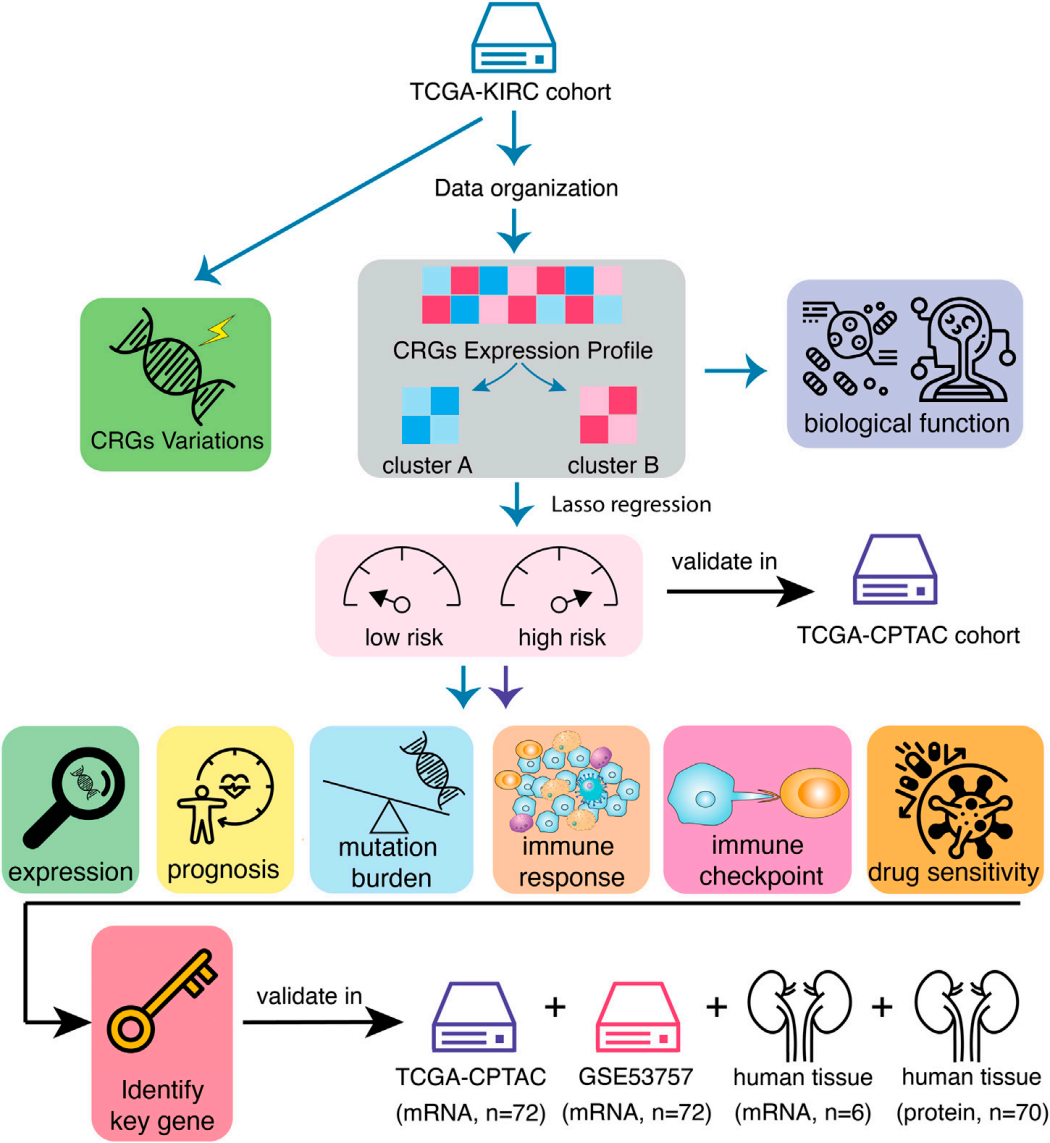
Workflow of data analysis in the study. Data of 541 samples from TCGA-KIRC cohort have been organized and the variations of calcium-related genes (CRGs) have been explored. Then, KIRC patients have been divided into two clusters according to CRGs expression profile. The differences of prognosis and biological functions have been investigated based on CRG clusters. In order to screen the most prognostic-related genes, univariate Cox regression and LASSO analysis were performed and the KIRC patients have been divided into CRGs-related high-risk and low-risk subtypes. These results have been validated in another mRNA cohort, TCGA-CPTAC. Immune landscapes, including tumor mutation burden, immune cell infiltration, immune checkpoint expression as well as the sensitivity of immunotherapies, have been tested in distinct risk subtypes. Finally, key CRG that most associated with prognosis in KIRC tumors has been identified and validated.

The flowchart of the entire study is summarized in Figure 1.

### 3.1 Genetic landscape of the CRGs in kidney renal clear cell carcinoma tumors

**FIGURE 2:**
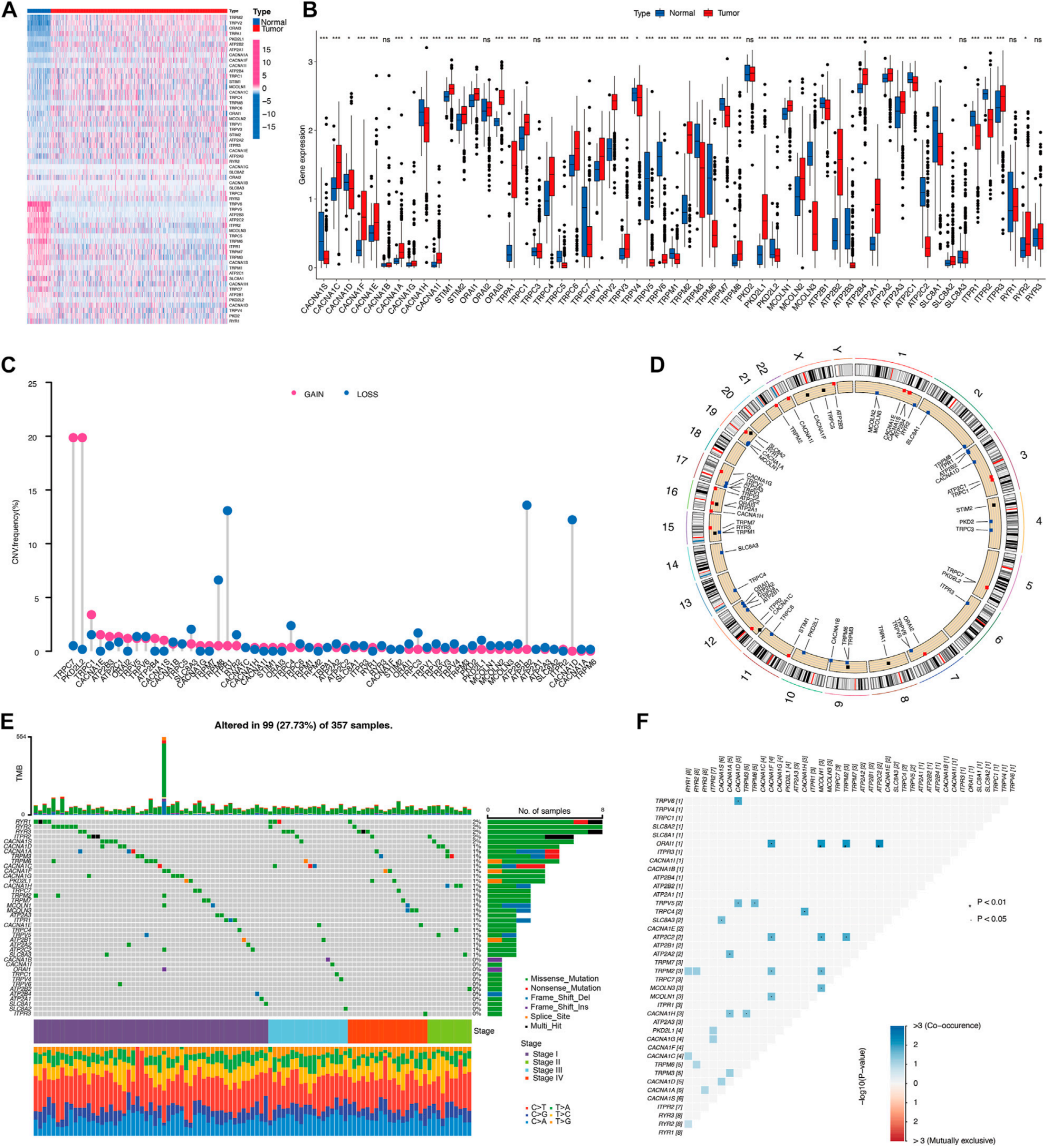
Landscape of calcium-related genes (CRGs) in TCGA cohort. **(A,B)** The differential expression of 58 CRGs in KIRC and normal tissue samples. **(C)** Copy number variation (CNV) status of 58 CRGs. **(D)** The location of CNV in chromosome. **(E)** Differential somatic mutations of CRGs. **(F)** Co-occurrence of mutations of CRGs.

A total of six subtypes of calcium channels, pumps, exchangers were summarized and 58 CRGs were selected to identify the expression in 541 KIRC cases compared to 72 normal cases in TCGA cohorts. It was obvious that the majority of selected CRGs were differentially expressed between normal and tumor cases (Figures 2A, B). VCGG channels, including CACNA1C, CACNA1D, CACNA1F, CACNA1E, CACNA1A, CACNA1G, CACNA1I, showed significantly higher expression in KIRC than normal cases, whereas CACNA1S and CACNA1H showed higher expression in normal than tumor cases (Figure 2B). Most of the CRAC channels had remarkably increased expression levels in KIRC (Figure 2B). TRP channels, including TRPA1, TRPC1, TRPC3, TRPC4, TRPC6, TRPV1-4, TRPM2, TRPM8, PKD2L1, MCOLN1, MCOLN2 had simultaneous high expression levels in tumor cases, while TRPC5, TRPC7, TRPV5, TRPV6, TRPM1, TRPM3, TRPM6, TRPM7, PKD2L2, MCOLN3 had high expression levels in normal cases (Figure 2B). Ca^2+^-ATPase pumps also showed different expression patterns (Figure 2B). Na^+^/Ca^2+^ exchangers 1 (SLC8A1) had significantly low expression in tumors, while Na^+^/Ca^2+^ exchangers 2 (SLC8A2) had the reverse result (Figure 2B). As a result, CRG expression could clearly distinguish KIRC patients from normal and tumor.

A copy number variation (CNV) is a loss or gain of a large region of tumor DNA which may activate oncogenes or deactivate tumor suppressors (Beroukhim et al., 2010). We therefore calculated and demonstrated a frequent alteration in 58 CRGs, with the most significant CNV depletion in TRPM8, ITPR1, ATP2B2, CACNA1D genes and the most significant CNV amplification in TRPC7 and PKD2L2 genes in KIRC tumors (Figure 2C). The location and mutation frequency of CNV of 58 CRGs on the chromosome were indicated in Figure 2D. In addition, somatic mutation analysis found that 99 out of 357 samples carried CRG mutations, representing a 27.73% mutation frequency (Figure 2E). KIRC stage IV showed the highest mutation frequency of 42.9%, while mutation frequency in stages I, II, III were 25.7%, 26.3%, 26.9%, respectively. Among these, RYR1-3 genes had the highest mutation frequency (Figure 2E). There is a high frequency of RYR2 mutations in various types of cancer in humans (Xu et al., 2021a). Mutations in RYR2 are associated with a number of immune-related pathways and are associated with a greater abundance of immune cell infiltration (Liu et al., 2021). Additionally, the frequencies of CRGs mutation co-occurrences were observed in KIRC (Figure 2F). Collectively, these results suggest distinct mRNA expression and variation of CRGs occurred in KIRC tumors.

### 3.2 Evaluation of prognosis, biological function, and immune infiltration of CRG clusters

**FIGURE 3:**
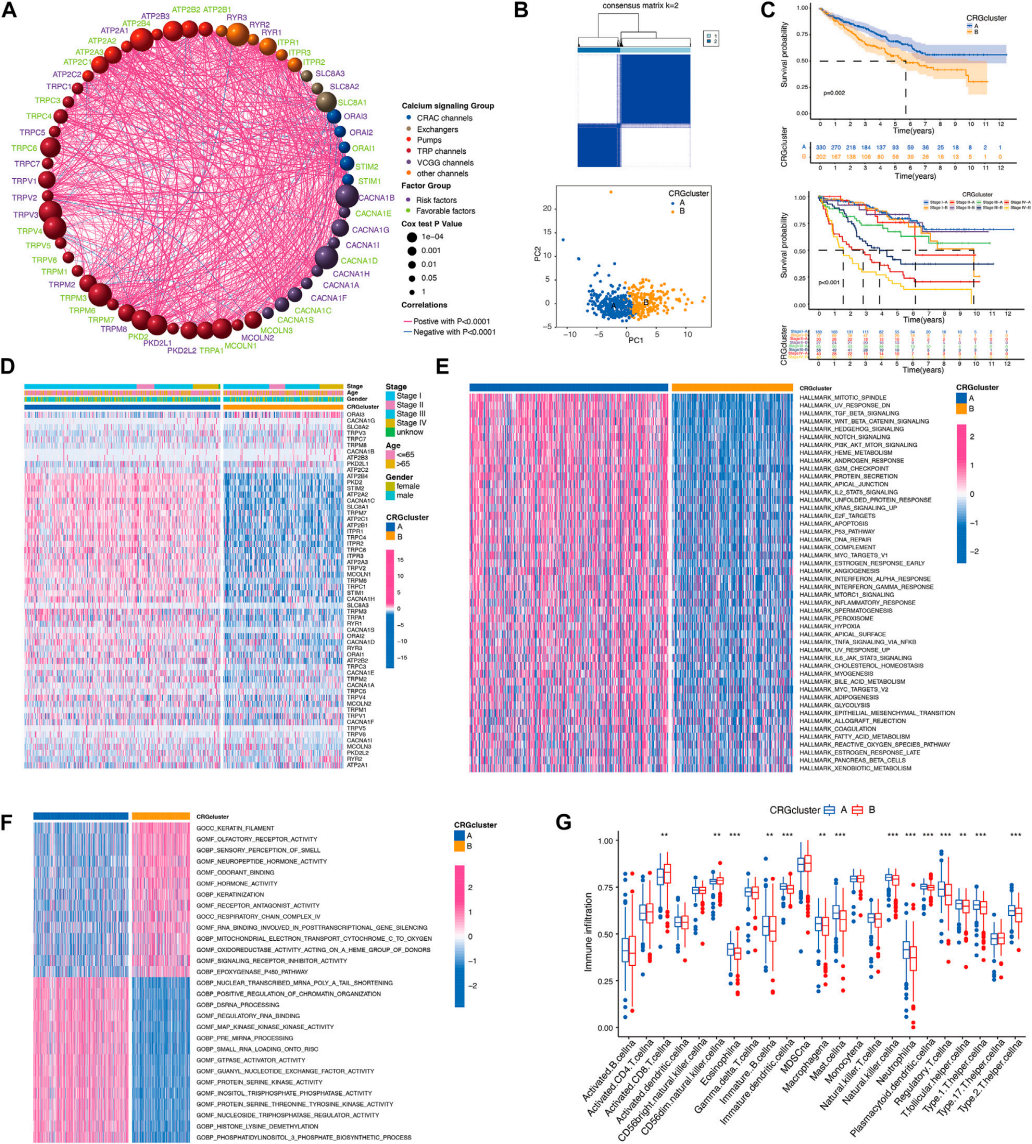
Evaluation of biological functions of CRGs. **(A)** The interactions between CRGs. The circle size represents the *p*-values of CRGs on the prognosis. Gene names with green color represent favorable factors for prognosis, and purple color represent risk factors for prognosis. The thickness of the lines represents the strength of correlation between CRGs. Negative and positive correlations were marked with blue and red, respectively. **(B)** The consensus matrixes of KIRC samples (*k* = 2). **(C)** Kaplan–Meier curves showed the overall survival between calcium clusters. **(D)** CRGs expression between calcium clusters. **(E,F)** HALLMARK and GO signature enrichment between calcium clusters. **(G)** Immune cell infiltration between calcium clusters.

Next, we explored the interactions, prognostic impact, and biological functions of the CRGs. As shown in Figure 3A, the 58 CRGs, divided into six groups (VGCC channels, CRAC channels, TRP channels, other channels, pumps, and exchangers), were interacted with each other. CRGs with favorable and unfavorable prognoses were negatively correlated (green and purple, respectively), such as the unfavorable factor ORAI3 has a negative correlation with favorable prognostic factors MCOLN3, TRPM8, and TRPC3 (Figure 3A). Based on the different CRG expression patterns, a cluster analysis was performed using the R package “ConsensusClusterPlus” (Wilkerson and Hayes, 2010). Two calcium-related clusters were identified and defined as CRG clusters A and B (Figure 3B). The prognostic analysis of these two clusters showed that cluster A yielded a better prognosis as compared to cluster B both in whole KIRC tumors (*p* = 0.002) or individual stage I, II, III, and IV tumors (*p* < 0.001, Figure 3C). We also used a heatmap to illustrate the expression pattern of the CRGs which showed a distinct expression pattern in these two clusters (Figure 3D).

In order to explore the differences in biological behaviors between the two clusters of CRGs, we conducted HALLMARK, KEGG, and GO-related GSVAs (Figures 3E, F; Supplementary Figure S1A). HALLMARK analysis revealed that KIRC patients in cluster B exhibited the enrichment of a wide range of processes including apoptosis, inflammatory response, hypoxia, glycolysis, fatty acid metabolism, etc. Cellular signaling such as TGF-beta signaling, hedgehog signaling, notch signaling, PI3K-AKT-mTOR signaling were also enriched in calcium cluster B (Figure 3E). GO analysis also showed a wide range of multiple biological process, molecular function, and cellular component in cluster B (Figure 3F). Then, we analyzed the immune cell infiltration characteristics between CRG cluster A and B, most of the immune cells, such as immature B cells, immature dendritic cells, macrophage, mast cells, nature killer (NK) cells, neutrophils, regulatory T cells (Tregs), and T helper cells, infiltrated more in calcium cluster B tumors, while CD8 positive cells and CD56dim NK cells infiltrated more in cluster A cells (Figure 3G). Taken together, these results indicated that CRGs are involved in various cellular functions including immune and inflammatory responses.

### 3.3 Development of the CRGs-related prognostic risk score

Univariate Cox regression and LASSO analysis were performed and 5 of the 58 CRGs were identified (Supplementary Figure S1B). The calcium-related prognostic signature was developed to assess the prognostic performance of these five genes (ORAI3, TRPV4, TRPM3, ATPRA1, and ITPR1). Predictive models were developed by combining the expression levels of five genes and categorizing patients in the TCGA cohort as high- or low-risk. Risk score = (0.31411 * ORAI3) + (−0.19961 * TRPV4) + (−0.25600 * TRPM3) + (0.39770 * ATPRA1) + (−0.28296 * ITPR1).

**FIGURE 4:**
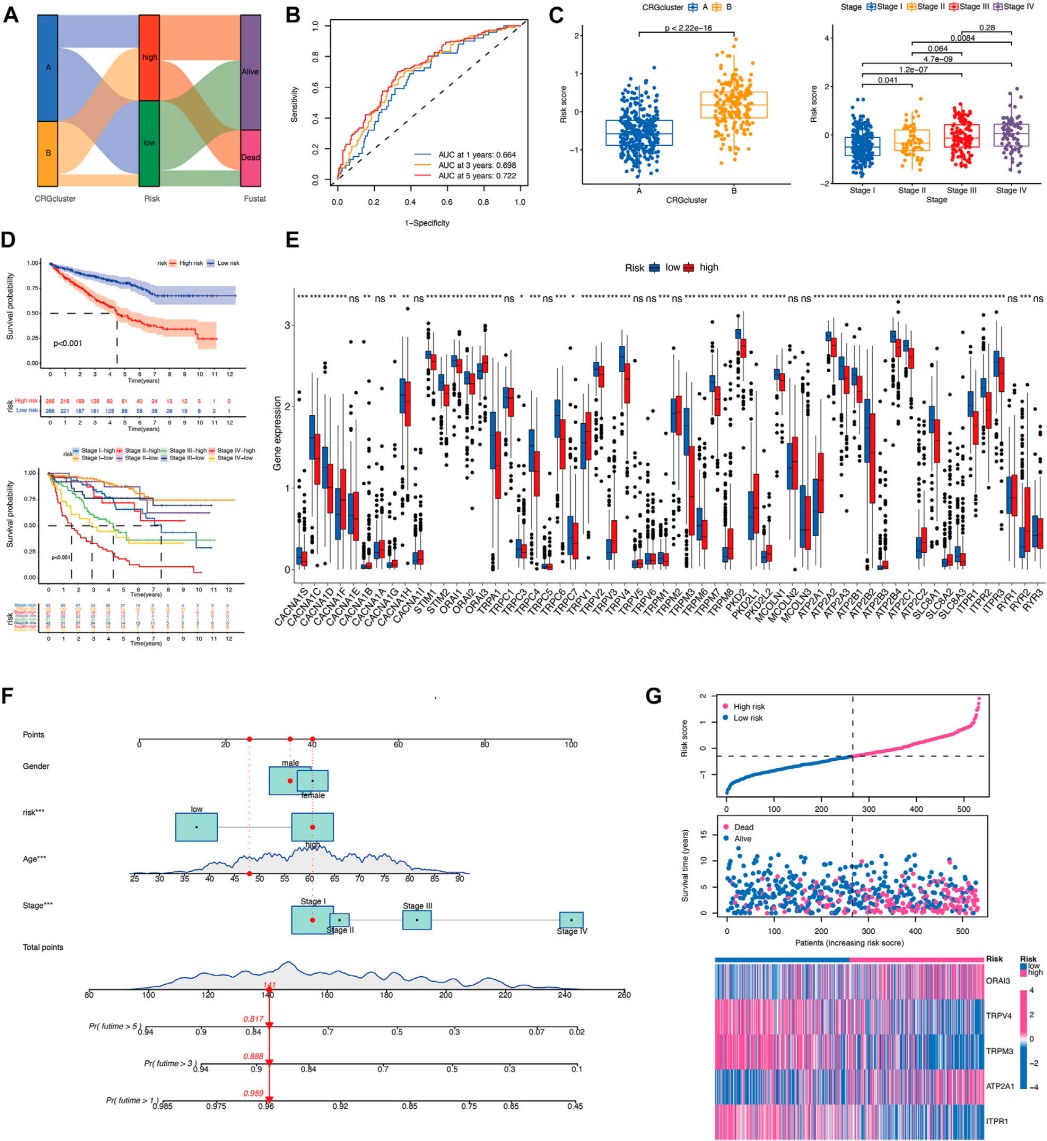
Development of the CRGs-related risk score in TCGA cohort. **(A)** The Sankey diagram demonstrates the distribution of patients with calcium clusters, CRGs-related risk score, and patient status. **(B)** ROC curve shows the good predictive value for development of CRGs-related risk score. **(C)** Risk score of KIRC patients in different calcium clusters and different disease stages. **(D)** Kaplan–Meier curves showed the overall survival between CRGs-related risk subtypes in TCGA cohort. **(E)** CRGs expression between CRGs-related risk subtypes. **(F)** The nomogram is created for calculating the total score of KIRC patients based on the values of each variable. **(G)** Five prognostic-related genes expression in order of increasing risk score.

In order to assess the importance of CRGs in patients with KIRC, we performed a personalized assessment. Sankey plot was used to visualize the distribution of CRG clusters, CRGs-related risk score, and patient status. Figure 4A shows that the majority of patients in cluster A had a lower risk score and better prognosis than those in cluster B. ROC curve in Figure 4B showed that the development of CRGs-related risk score exhibited a good predictive value. We also analyzed the role of risk score in the aspect of CRG clusters and disease stages in TCGA cohorts. KIRC patients in cluster B showed significantly high-risk score than that in cluster A (Figure 4C). Patients with stage III and IV had remarkably higher risk score than stage I and II (Figure 4C). Prognostic value showed that KIRC patients with high-risk score displayed poor prognosis (Figure 4D) which was also validated in TCGA-CPTAC database (Supplementary Figure S1C). The expression of 58 CRGs in distinct risk score groups can be visualized in Figure 4E. In addition, a nomogram was created to calculate the total score based on the values of each variable, including gender, age, risk score, and disease stage, for the overall survival prediction of KIRC patients (Figure 4F). The expression of these five gene based on risk core was shown in Figure 4G. Altogether, KIRC patients could be divided into distinct risk subtypes based on CRGs expression.

### 3.4 Evaluation of immune landscape of CRGs-related risk subtypes

**FIGURE 5:**
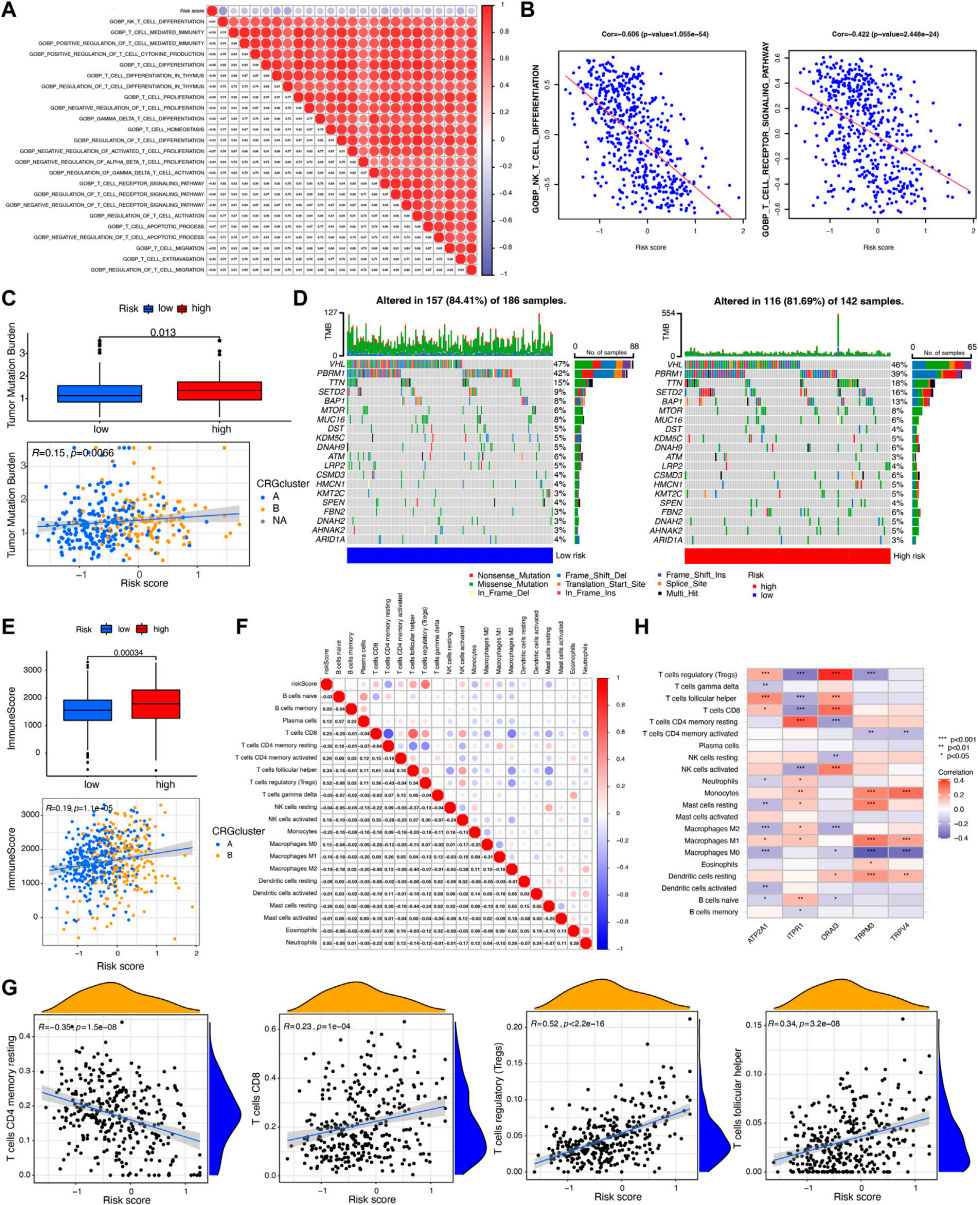
Evaluation of tumor mutation burden (TMB) and immune cell infiltration between CRGs-related risk subtypes. **(A,B)** The correlation between T cell-related immune pathways and CRGs-related risk score. **(C)** Differences in TMB between CRGs-related risk subtypes. **(D)** Genes with the highest mutation frequency in CRGs-related risk subtypes. **(E)** Differences in immune score between CRGs-related risk subtypes. **(F,G)** The correlation between 22 immune cell infiltration and CRGs-related risk score. **(H)** The correlation of five prognostic-related CRGs with immune cell infiltration.

In order to evaluate the immune landscape of CRGs-related risk subtypes, we first calculated the correlation between CRGs-related risk score and immune-related pathways. As shown in Figures 5A, B, CRGs-related risk score showed a significant negative correlation with T cell-related immune pathways such as T cell differentiation, T cell mediated immunity, T cell cytokine production, T cell proliferation and migration, and T cell receptor signaling pathway (correlation > 0.3, *p* < 0.05).

The tumor mutation burden (TMB) indicates how many changes a cancer cell has undergone genetically. Cancers with low tumor mutation burden decrease the chance to activate immune system, whereas cancers with high tumor mutation burden increase the chance to activate immune response (Fusco et al., 2021). In this study, we then tested the TMB in distinct risk score groups in TCGA cohort. Figure 5C showed that patients in high-risk score groups revealed relatively higher TMB than that in low-risk score groups, but the correlation is weak (R = 0.15). Different somatic mutations of the top 20 driver genes with the highest mutation frequency were also analyzed. Figure 5D showed that the mutations were frequently observed both in low- and high-risk score groups, which means immune cells in distinct risk groups have the similar chance to identify cancer cells.

**FIGURE 6:**
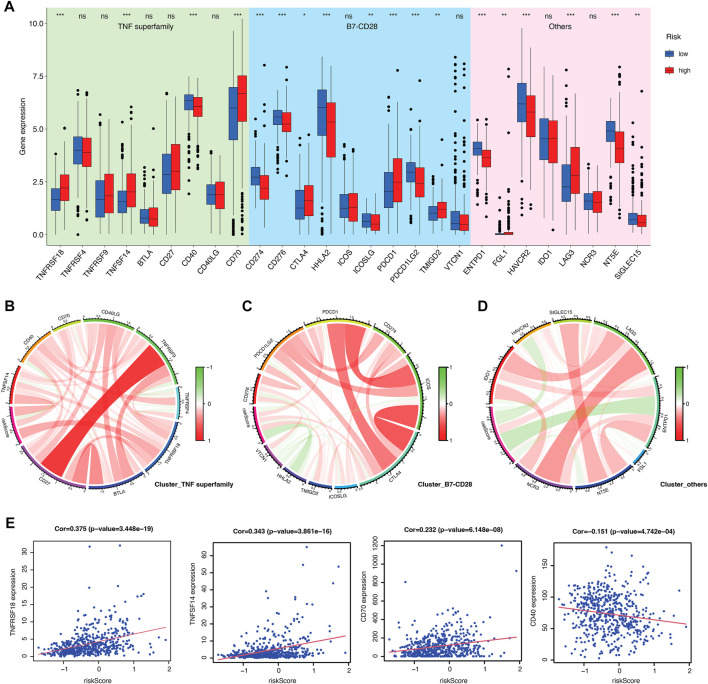
Evaluation of immune checkpoints between CRGs-related risk subtypes. **(A)** Gene expression of immune checkpoints between CRGs-related risk subtypes. **(B–D)** Correlation of immune checkpoint members of TNF superfamily **(B)**, B7-CD28 superfamily **(C)**, and others **(D)**.**(E)** The correlation of specific immune checkpoint members with risk score.

Furthermore, immune characterization was also investigated in CRGs-related risk groups. CRGs-related risk score was positively correlated with immune score (Figure 5E), and strongly associated with regulatory T cells (R = 0.52), follicular T helper cells (R = 0.34), CD8 positive T cells (R = 0.23), and CD4 resting memory T cells (R = −0.35) (Figures 5F, G). The relationship of immune cell infiltration with each risk genes was additionally shown in Figure 5H. High risk groups showed high expression of ORAI3 and ATP2A1, which positively related to regulatory T cells and follicular T cells, and negatively correlated to M2 and M0 macrophages (Figure 5H). Moreover, low expression of TRPV4, TRPM3, and ITPR1 were found in high-risk groups with a negative association with regulatory T cells, follicular T cells, and a positive association with monocytes and M1 macrophages (Figure 5H). In addition, the expression of 27 immune checkpoint molecules, including the B7-CD28 superfamily, TNF superfamily, and other molecules was tested in CRGs-related risk groups (Figure 6A). It is interesting that CD274 (PD-L1) had a low expression level in high-risk group, whereas PDCD1 (PD-1) and CTLA-4 appeared high level of expression in high-risk group (Figure 6A). The correlation between each superfamily was shown in Figures 6B–D. Collectively, high-risk CRG subtype showed relatively immunosuppression in KIRC tumors due to high infiltration of Tregs and high expression of PD-1 and CTLA-4. In the TNF superfamily, CRGs-related risk score positively correlated with TNFRSF18, TNFSF14, and CD70 expression, while negatively correlated with CD40 expression (Figure 6E). Inhibiting or stimulating TNF superfamily signaling pathways may have therapeutic benefits for cancer patients (Vanamee and Faustman, 2018).

### 3.5 Evaluation of chemotherapy efficacy based on CRGs-related risk score

**FIGURE 7:**
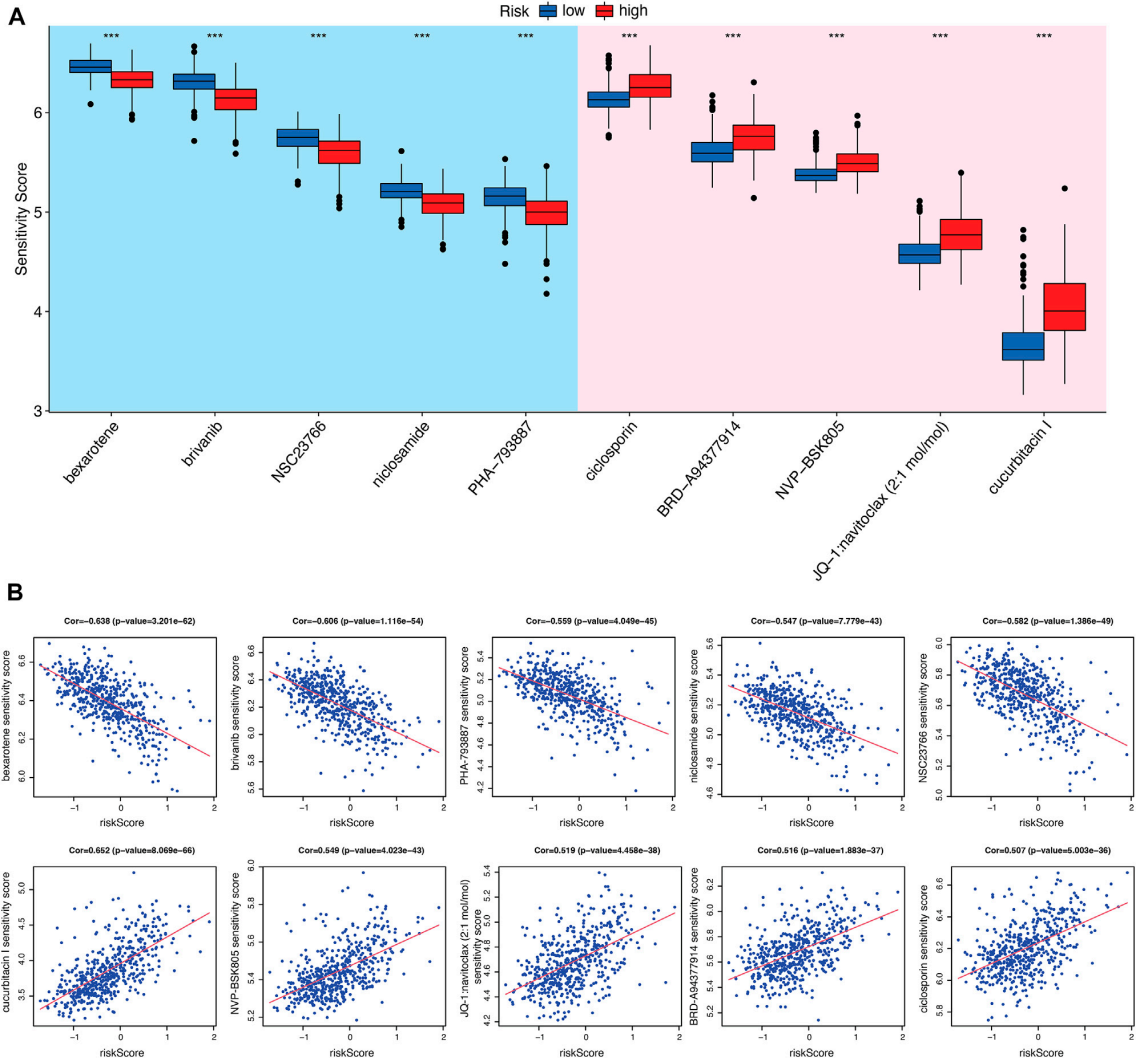
Evaluation of chemo-/immuno-therapy efficacy between CRGs-related risk subtypes. **(A)** The difference of multiple agent sensitivity between CRGs-related risk subtypes. **(B)** The correlation of these agent with risk score.

In order to determine the value of risk score in predicting the clinical therapeutic efficacy of KIRC, we examined the sensitivity of several agents between the CRGs-related subtypes. The criteria for screening chemotherapies were as follows: 1) *p*-value is less than 0.001; 2) the correlation is greater than or less than 0.5; 3) the value of log_2_FC is from high to low. As a result, five drugs that were more sensitive to high-risk subtypes and five drugs that were more sensitive to low-risk subtypes were selected (Figures 7A, B). These results indicate that the immune checkpoints inhibition combined with these chemo-drugs would be a promising strategy for treating KIRC patients in distinct CRGs-related risk subtypes.

### 3.6 TRPM3 was identified as the prognostic key gene

**FIGURE 8:**
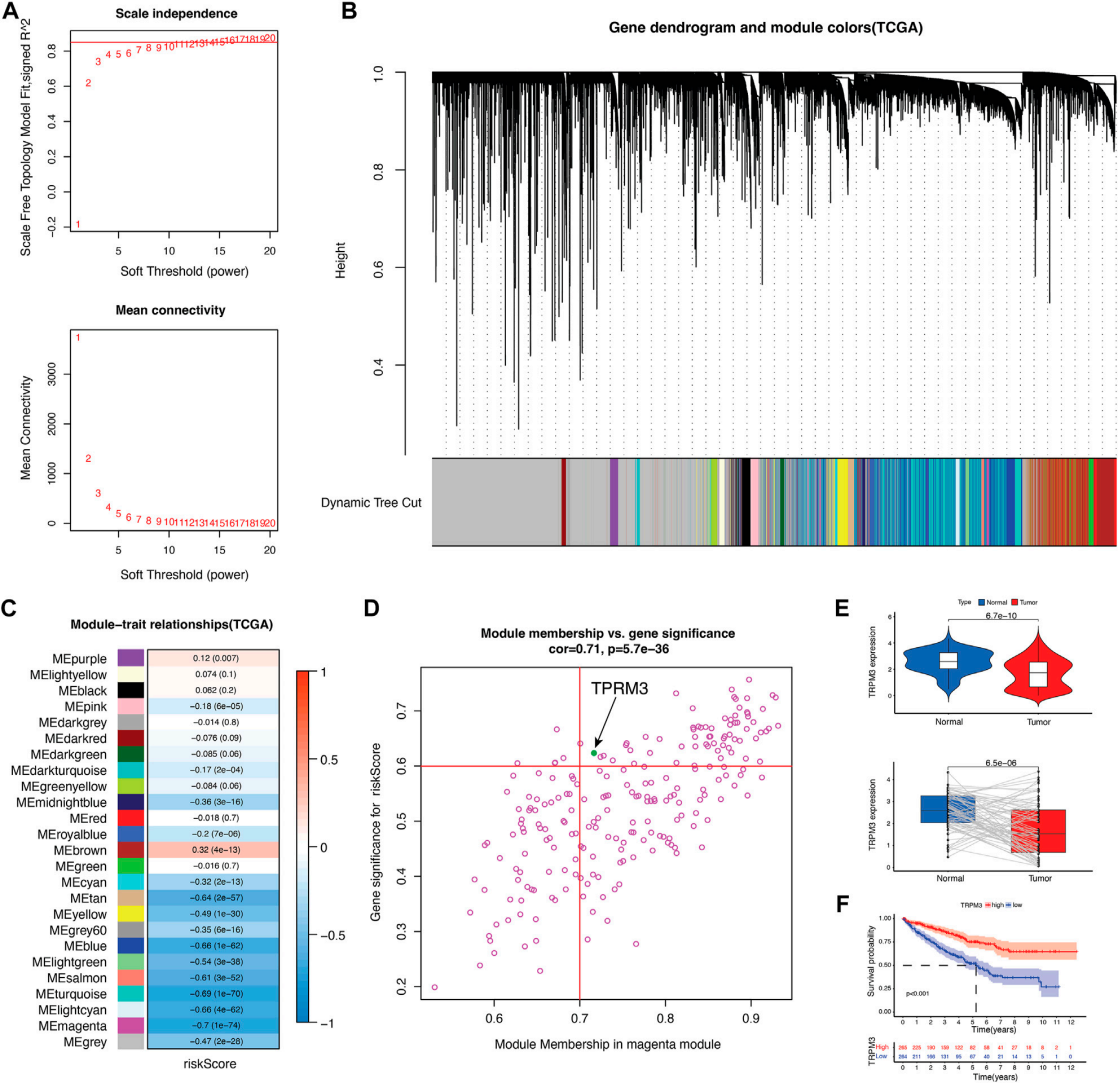
Identify key CRG in KIRC. **(A)** scale-free co-expression network. **(B)** 25 gene modules were obtained in a dendrogram. **(C)** The correlation coefficient and corresponding *p*-value between each gene module and risk score. **(D)** Scatter plot of module eigengenes in the magenta module. **(E)** TRPM3 mRNA expression between tumor and normal tissues as well as paired tumor-normal tissues in TCGA cohort (*n* = 72). **(F)** Kaplan–Meier curves showed overall survival difference between high and low TRPM3 expression groups in TCGA cohort.

An analysis of the key genes was further enhanced by building a gene co-expression network based on WGCNA to identify modules that have the strongest correlation with risk scores. In Figure 8A, we constructed a scale-free co-expression network based on the number of 15 as the appropriate soft threshold. The final result was 25 modules (Figure 8B). Figures 8C, D show that magenta module was most negatively correlated with risk score. To identify the key CRGs that associated with prognosis, we crossed the five genes with genes of the magenta module, and TRPM3 were obtained. To validate the importance of TRPM3 in KIRC tumors, mRNA expression and prognostic analysis were applied. There were significant differences between KIRC and normal cases in the expression of TRPM3 (Figure 8E, Supplementary Figure S1D). The same result was also found in KIRC-normal paired patients (Figure 8E). Meanwhile, patients with lower expression of TRPM3 were related to poor prognosis (Figure 8F, Supplementary Figure S1E).

**FIGURE 9:**
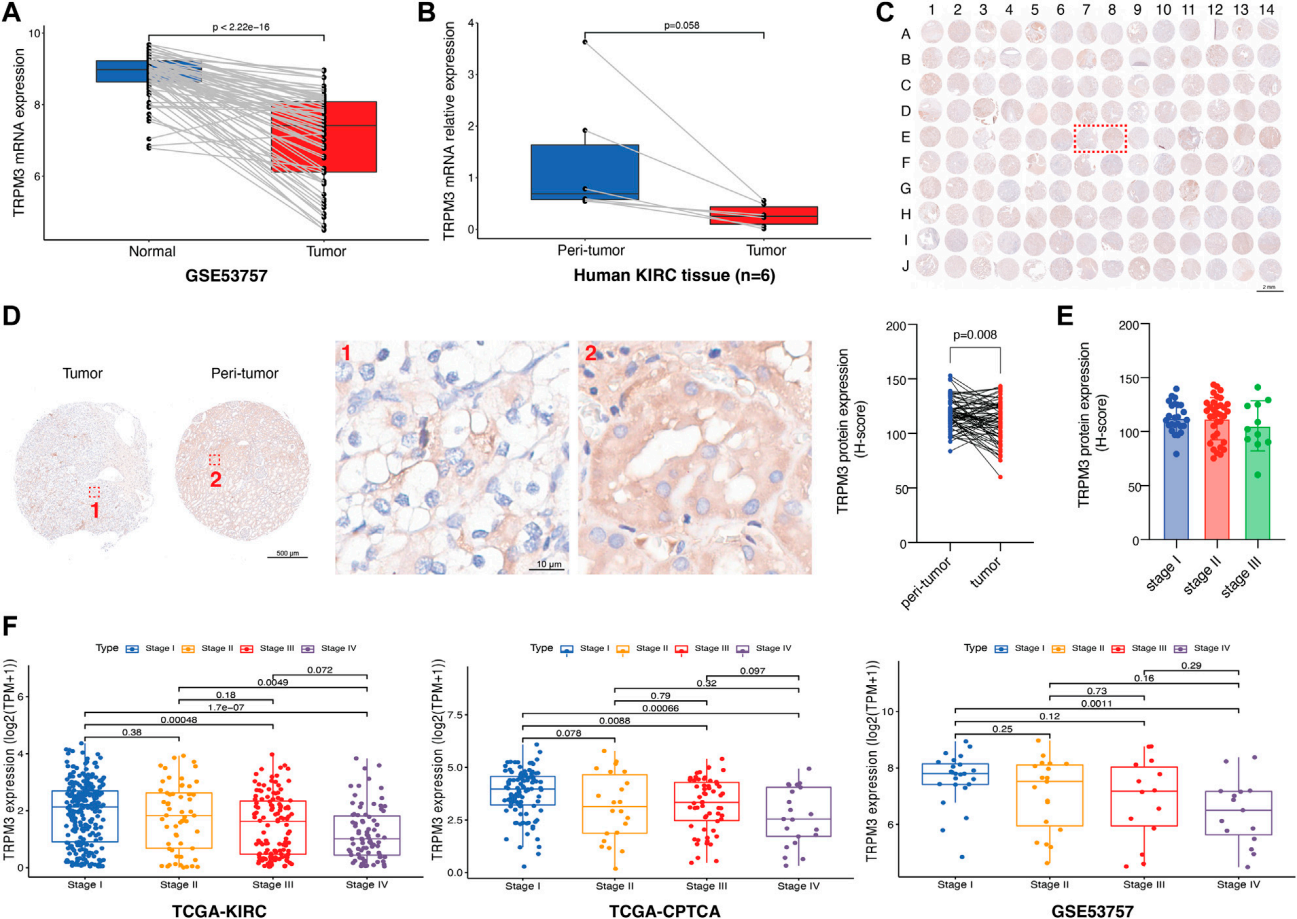
Validation of TRPM3 in KIRC. **(A)** TRPM3 mRNA expression between paired tissues in GSE53757 cohort (*n* = 72). **(B)** TRPM3 mRNA expression between human KIRC paired tissues (*n* = 6). **(C)** Tumor microarray of 70 KIRC paired tissues stained for TRPM3 protein. **(D)** TRPM3 protein expression between paired tissues in tissue microarray [Representative images are the ones inside the red wireframe in **(C)**]. **(E)** TRPM3 protein expression between different KIRC stages. **(F)** TRPM3 mRNA expression between different KIRC stages in TCGA-KIRC, TCGA-CPTAC, and GSE53757 datasets.

To validate the reliability and authenticity of the results in TCGA, we analyzed the GSE53757 dataset and human KIRC tissues. The results showed that TRPM3 mRNA expression was lower in tumor tissues relative to peri-tumor tissues (Figures 9A, B). TRPM3 protein expression was validated in 70 paired KIRC patients and found the same trend (Figures 9C, D). TRPM3 protein and mRNA expression in different stages of disease has also been identified. The TRPM3 protein found no significant expression between stage I to III (Figure 9E), however, mRNA expression of TRPM3 was remarkably higher in KIRC stage I than stage III-IV in TCGA and GSE53757 datasets (Figure 9F). Based on these findings, TRPM3 deficiency may play an important role in KIRC tumor progression, providing a therapeutic strategy for KIRC treatment.

## 4 Discussion

Renal cell carcinoma is a malignant tumor accounting for nearly 90% of renal tumors and 3% of adult tumors (Hsieh et al., 2017). KIRC is the most typical subtype of renal cell carcinoma and approximately 30% of patients are diagnosed in the advanced stage (Tan et al., 2021). The prognosis of KIRC, compared to other histological subtypes, was generally worse (Xu et al., 2021b). Current treatment strategy for KIRC is limited. Despite efforts made with targeted therapies, several limitations such as low rates of durable response, resistance, and side effects of these agents make clear the need for novel therapies (Brown et al., 2020). The intracellular Ca^2+^ signaling pathway is a crucial component of multiple cellular processes. Ca^2+^ homeostasis dysregulation is implicated in driving the proliferative, migrating, invading, and metastatic processes of cancer (Chen et al., 2013). However, little is known about how calcium signaling functions in KIRC tumors. In order to evaluate the expression pattern, immune characteristics, and clinical relevance of CRGs, we collected and consolidated transcriptomic data from a large number of studies. The purpose of this study is to perform a comprehensive analysis of the potential roles of CRGs in the landscape of immunity within KIRC microenvironments, which will provide promising strategies for therapeutic intervention.

In our study, we first analyzed the expression patterns of 58 CRGs in KIRC tumors compared to normal cases and found that most of the CRGs had a distinct expression pattern which indicated that the expression of CRGs could completely distinguish KIRC tumor patients from normal cases. However, it was surprising that, CACNA1B, ORAI2, TPRC3, PKD2, SLC8A3, RYR1, and RYR3, seemed to be unaltered in these groups. The CNV of CRGs was then summarized in KIRC tumors, significantly amplification was found only in TPRC7 and PKD2L2 genes. However, these two genes in KIRC tumors have not yet been studied. TRPM8, ITPR1, ATP2B2, and CACNA1D genes showed the most significant CNV depletion in our study. Typically, ITPR1 as an autophagy sensor is downregulated in KIRC tumors, but has been reported to protect renal carcinoma cells from NK-mediated killing (Messai et al., 2015).

Next, we found the signature of 58 CRGs could well predict the prognosis of KIRC patients. Accordingly, calcium cluster A patients had a better prognosis with a median survival time of over 12 years, whereas calcium cluster B patients had a worse prognosis with a median survival time of only 5 years. It suggests that calcium-related gene signatures are a good predictor of KIRC patients. Furthermore, biological functions of CRGs between calcium cluster A and B were also analyzed and a wide range of processes including apoptosis, inflammatory response, hypoxia, glycolysis, fatty acid metabolism was observed in calcium cluster B. In terms of the efficiency of immunotherapy in renal cell carcinoma (Brown et al., 2020), we then focused the role of CRGs on immune and inflammatory responses.

Then, we constructed a prognostic signature based on five calcium-related genes (ORAI3, TRPV4, TRPM3, ATPRA1, and ITPR1) by using univariate Cox regression and LASSO analysis, and then divided KIRC patients into high- and low-risk groups. Overexpression of ORAI3 has been observed in various cancer cells as compared to normal cells. A number of cancer hallmarks are supported by overexpression of ORAI3, including cell cycle progression, proliferation, migration, and resistance to apoptosis (Sanchez-Collado et al., 2021). TRPV4 and TRPM3 have been shown positively related to tumor progression (Li et al., 2020; Zhang et al., 2021). However, it is interesting that these two genes have a negative association with KIRC risk score, which needs further investigation.

Immune cells infiltrating tumors and immune checkpoints within the tumor microenvironment may be predictive of cancer prognosis and treatment response. Calcium signaling also have been reported to be related to T cell activation and proliferation (Vaeth et al., 2020; Acharya et al., 2021). There was a strong positive correlation between the high-risk group and Treg infiltration, which are involved in tumor development and progression by inhibiting antitumor immunity (Ohue and Nishikawa, 2019). However, the roles of calcium signaling in regulating Tregs are unclear, which needs further investigation. The relationship between CRGs-related risk score and immune checkpoints also suggested that calcium may play a suppressive role in the anti-tumor immune responses due to the negative association with PD-1, CTLA-4, and other inhibitory immune checkpoints. A positive correlation was found between CRGs-related risk score and TNFRSF18, TNFSF14, and CD70, whereas a negative correlation was found between CRGs-related risk score and CD40 expression. A high level of constitutive TNSRSF18 expression is found on Tregs. It appears that inhibition of TNFRSF18 with its ligand TNFSF18 may result in the biological outcome of inhibiting Treg function while activating CD8^+^ T effectors (Knee et al., 2016). TNFSF14 is a protein primarily expressed on activated T cells, activated NK cells, and immature dendritic cells. However, only when the TNFSF14 molecule is delivered to or expressed within tumors can it exert the synergistic effect with immune checkpoint inhibitors, thereby activating the ability of immune cells to kill tumor cells (Skeate et al., 2020). CD70 is also implicated in tumor cell and regulatory T cell survival through interaction with its ligand, CD27. Blocking CD70/CD27 axis can abolish immune escape by T cell apoptosis, T cell exhaustion and Treg survival, therefore inhibiting tumor proliferation (Jacobs et al., 2015). CD40 is a cell-surface member of the TNF receptor superfamily. Upon activation, CD40 promotes antitumor T cell activation and reeducates macrophages to destroy tumor stroma (Vonderheide, 2020). Collectively, modulation of calcium signaling together with immunotherapy would be a promising strategy for treating KIRC patients.

Although the tumor mutation burden in renal cell carcinoma is relatively low, there are a large number of genomic alteration that may account for the neoantigens required for the T cell anti-tumor response (Turajlic et al., 2017; Brown et al., 2020). Immunotherapy mono-treatment or combined with chemotherapy are very prevalent nowadays in KIRC patients. Therefore, in our study, we analyzed the sensitivity of several clinical therapeutic agents between CRGs-related risk subtypes in KIRC. Bexarotene (retinoid X receptors agonist), brivanib (ATP-competitive VEGFR2 inhibitor), NSC23766 (Rac GTPase inhibitor), niclosamide (STAT3 inhibitor) and PHA-793887 (ATP-competitive CDK inhibitor) were more sensitive to high-risk subtype. In contrast, ciclosporin (immunosuppressant), NVP-BSK805 (JAK2 inhibitor), navitoclax (Bcl-2 inhibitor) and cucurbitacin I (JAK2/STAT3 inhibitor) were more sensitive to the low-risk subtype. These results suggest that immunotherapy with immune checkpoint inhibitors would be more effective in combination with these chemotherapeutic agents for the treatment of KIRC.

In order to find the CRGs that most associated with prognosis in KIRC tumors, we screened out key adverse prognostic regulators. Surprisingly, our analysis showed that TRPM3 was a key CRG that related to poor prognosis. The TRPM belongs to a non-selective cation channel family composed of eight members (Jimenez et al., 2020), which have a ubiquitous expression in tissues that associated with participation in health and diseases (Fonfria et al., 2006). Recent studies have shown that activation of TRPM3 induces interleukin 8 secretion through activation of c-Jun and transcription factor 2, explaining the involvement of TRPM3 activation in inflammatory response (Rubil et al., 2018). However, little is known about the role of TRPM3 in KIRC tumors, which needs further investigation.

## Data Availability

The datasets presented in this study can be found in online repositories. The names of the repository/repositories and accession number(s) can be found in the article/Supplementary Material.

## References

[B1] Acharya T. K. Tiwari A. Majhi R. K. Goswami C. (2021). TRPM8 channel augments T-cell activation and proliferation. Cell Biol. Int. 45, 198–210. 10.1002/cbin.11483 33090595

[B2] Beroukhim R. Mermel C. H. Porter D. Wei G. Raychaudhuri S. Donovan J. (2010). The landscape of somatic copy-number alteration across human cancers. Nature 463, 899–905. 10.1038/nature08822 20164920 PMC2826709

[B3] Bray F. Ferlay J. Soerjomataram I. Siegel R. L. Torre L. A. Jemal A. (2018). Global cancer statistics 2018: GLOBOCAN estimates of incidence and mortality worldwide for 36 cancers in 185 countries. Ca. Cancer J. Clin. 68, 394–424. 10.3322/caac.21492 30207593

[B4] Brown L. C. Desai K. Zhang T. Ornstein M. C. (2020). The immunotherapy landscape in renal cell carcinoma. BioDrugs 34, 733–748. 10.1007/s40259-020-00449-4 33048299

[B5] Chen Y.-F. Chen Y.-T. Chiu W.-T. Shen M.-R. (2013). Remodeling of calcium signaling in tumor progression. J. Biomed. Sci. 20, 23. 10.1186/1423-0127-20-23 23594099 PMC3639169

[B6] Chen Y.-Y. Hu H. H. Wang Y. N. Liu J. R. Liu H. J. Liu J. L. (2020). Metabolomics in renal cell carcinoma: From biomarker identification to pathomechanism insights. Arch. Biochem. Biophys. 695, 108623. 10.1016/j.abb.2020.108623 33039388

[B7] Fonfria E. Murdock P. R. Cusdin F. S. Benham C. D. Kelsell R. E. McNulty S. (2006). Tissue distribution profiles of the human TRPM cation channel family. J. Recept. Signal Transduct. Res. 26, 159–178. 10.1080/10799890600637506 16777713

[B8] Fusco M. J. West H. Jack Walko C. M. (2021). Tumor mutation burden and cancer treatment. JAMA Oncol. 7, 316. 10.1001/jamaoncol.2020.6371 33331847

[B9] Goldman M. J. Craft B. Hastie M. Repecka K. McDade F. Kamath A. (2020). Visualizing and interpreting cancer genomics data via the Xena platform. Nat. Biotechnol. 38, 675–678. 10.1038/s41587-020-0546-8 32444850 PMC7386072

[B10] Ha Y.-S. Kim Y. Y. Yu N. H. Chun S. Y. Choi S. H. Lee J. N. (2018). Down-regulation of transient receptor potential melastatin member 7 prevents migration and invasion of renal cell carcinoma cells via inactivation of the Src and Akt pathway. Investig. Clin. Urol. 59, 263–274. 10.4111/icu.2018.59.4.263 PMC6028469 29984342

[B11] Hänzelmann S. Castelo R. Guinney J. (2013). GSVA: gene set variation analysis for microarray and RNA-seq data. BMC Bioinforma. 14, 7. 10.1186/1471-2105-14-7 PMC3618321 23323831

[B12] Hsieh J. J. Purdue M. P. Signoretti S. Swanton C. Albiges L. Schmidinger M. (2017). Renal cell carcinoma. Nat. Rev. Dis. Prim. 3, 17009. 10.1038/nrdp.2017.9 28276433 PMC5936048

[B13] Ibrahim S. Dakik H. Vandier C. Chautard R. Paintaud G. Mazurier F. (2019). Expression profiling of calcium channels and calcium-activated potassium channels in colorectal cancer. Cancers (Basel) 11, 561. 10.3390/cancers11040561 31010205 PMC6521016

[B14] Inamura K. (2017). Renal cell tumors: Understanding their molecular pathological epidemiology and the 2016 WHO classification. Int. J. Mol. Sci. 18, E2195. 10.3390/ijms18102195 PMC5666876 29053609

[B15] Jacobs J. Deschoolmeester V. Zwaenepoel K. Rolfo C. Silence K. Rottey S. (2015). CD70: An emerging target in cancer immunotherapy. Pharmacol. Ther. 155, 1–10. 10.1016/j.pharmthera.2015.07.007 26213107

[B16] Jimenez I. Prado Y. Marchant F. Otero C. Eltit F. Cabello-Verrugio C. (2020). TRPM channels in human diseases. Cells 9, 2604. 10.3390/cells9122604 33291725 PMC7761947

[B17] Jonasch E. Gao J. Rathmell W. K. (2014). Renal cell carcinoma. BMJ 349, g4797. 10.1136/bmj.g4797 25385470 PMC4707715

[B18] Kim J.-H. Lkhagvadorj S. Lee M. R. Hwang K. H. Chung H. C. Jung J. H. (2014). Orai1 and STIM1 are critical for cell migration and proliferation of clear cell renal cell carcinoma. Biochem. Biophys. Res. Commun. 448, 76–82. 10.1016/j.bbrc.2014.04.064 24755083

[B19] Klatte T. Rossi S. H. Stewart G. D. (2018). Prognostic factors and prognostic models for renal cell carcinoma: a literature review. World J. Urol. 36, 1943–1952. 10.1007/s00345-018-2309-4 29713755

[B20] Knee D. A. Hewes B. Brogdon J. L. (2016). Rationale for anti-GITR cancer immunotherapy. Eur. J. Cancer 67, 1–10. 10.1016/j.ejca.2016.06.028 27591414

[B21] Kristensen K. B. Habel L. A. Gagne J. J. Friis S. Andersen K. K. Hallas J. (2020). Risk of renal cell carcinoma associated with calcium channel blockers: A nationwide observational study focusing on confounding by indication. Epidemiology 31, 860–871. 10.1097/EDE.0000000000001256 32897909

[B22] Li W. Yang F. Q. Sun C. M. Huang J. H. Zhang H. M. Li X. (2020). circPRRC2A promotes angiogenesis and metastasis through epithelial-mesenchymal transition and upregulates TRPM3 in renal cell carcinoma. Theranostics 10, 4395–4409. 10.7150/thno.43239 32292503 PMC7150475

[B23] Li F. Qi B. Yang L. Wang B. Gao L. Zhao M. (2022). CHI3L1 predicted in malignant entities is associated with glioblastoma immune microenvironment. Clin. Immunol. 245, 109158. 10.1016/j.clim.2022.109158 36244672

[B24] Liu Z. Liu L. Jiao D. Guo C. Wang L. Li Z. (2021). Association of RYR2 mutation with tumor mutation burden, prognosis, and antitumor immunity in patients with esophageal adenocarcinoma. Front. Genet. 12, 669694. 10.3389/fgene.2021.669694 34079583 PMC8166246

[B25] Maeser D. Gruener R. F. Huang R. S. (2021). oncoPredict: an R package for predicting *in vivo* or cancer patient drug response and biomarkers from cell line screening data. Brief. Bioinform. 22, bbab260. 10.1093/bib/bbab260 34260682 PMC8574972

[B26] Messai Y. Noman M. Z. Janji B. Hasmim M. Escudier B. Chouaib S. (2015). The autophagy sensor ITPR1 protects renal carcinoma cells from NK-mediated killing. Autophagy 0, 00. 10.1080/15548627.2015.1017194 25714778

[B27] Nagashima K. Sato Y. (2017). Information criteria for Firth’s penalized partial likelihood approach in Cox regression models. Stat. Med. 36, 3422–3436. 10.1002/sim.7368 28608396 PMC6084330

[B28] Ohue Y. Nishikawa H. (2019). Regulatory T (Treg) cells in cancer: Can Treg cells be a new therapeutic target? Cancer Sci. 110, 2080–2089. 10.1111/cas.14069 31102428 PMC6609813

[B29] Petejova N. Martinek A. (2016). Renal cell carcinoma: Review of etiology, pathophysiology and risk factors. Biomed. Pap. Med. Fac. Univ. Palacky. Olomouc Czech. Repub. 160, 183–194. 10.5507/bp.2015.050 26558360

[B30] Prakriya M. Lewis R. S. (2015). Store-operated calcium channels. Physiol. Rev. 95, 1383–1436. 10.1152/physrev.00020.2014 26400989 PMC4600950

[B31] Ren J. Yuan Q. Liu J. Zhong L. Li H. Wu G. (2022). Identifying the role of transient receptor potential channels (TRPs) in kidney renal clear cell carcinoma and their potential therapeutic significances using genomic and transcriptome analyses. BMC Med. Genomics 15, 156. 10.1186/s12920-022-01312-x 35831825 PMC9277847

[B32] Rubil S. Lesch A. Mukaida N. Thiel G. (2018). Stimulation of transient receptor potential M3 (TRPM3) channels increases interleukin-8 gene promoter activity involving AP-1 and extracellular signal-regulated protein kinase. Cytokine 103, 133–141. 10.1016/j.cyto.2017.09.020 28982580

[B33] Sanchez-Collado J. Jardin I. Lopez J. J. Ronco V. Salido G. M. Dubois C. (2021). Role of Orai3 in the pathophysiology of cancer. Int. J. Mol. Sci. 22, 11426. 10.3390/ijms222111426 34768857 PMC8584145

[B34] Skeate J. G. Otsmaa M. E. Prins R. Fernandez D. J. Da Silva D. M. Kast W. M. (2020). TNFSF14: LIGHTing the way for effective cancer immunotherapy. Front. Immunol. 11, 922. 10.3389/fimmu.2020.00922 32499782 PMC7243824

[B35] Tan P. Chen H. Huang Z. Huang M. Du Y. Li T. (2021). MMP25-AS1/hsa-miR-10a-5p/SERPINE1 axis as a novel prognostic biomarker associated with immune cell infiltration in KIRC. Mol. Ther. Oncolytics 22, 307–325. 10.1016/j.omto.2021.07.008 34553021 PMC8426181

[B36] Tibshirani R. (1997). The lasso method for variable selection in the Cox model. Stat. Med. 16, 385–395. 10.1002/(sici)1097-0258(19970228)16:4<385::aid-sim380>3.0.co;2-3 9044528

[B37] Turajlic S. Litchfield K. Xu H. Rosenthal R. McGranahan N. Reading J. L. (2017). Insertion-and-deletion-derived tumour-specific neoantigens and the immunogenic phenotype: a pan-cancer analysis. Lancet. Oncol. 18, 1009–1021. 10.1016/S1470-2045(17)30516-8 28694034

[B38] Vaeth M. Kahlfuss S. Feske S. (2020). CRAC channels and calcium signaling in T cell-mediated immunity. Trends Immunol. 41, 878–901. 10.1016/j.it.2020.06.012 32711944 PMC7985820

[B39] Vanamee É. S. Faustman D. L. (2018). Structural principles of tumor necrosis factor superfamily signaling. Sci. Signal. 11, eaao4910. 10.1126/scisignal.aao4910 29295955

[B40] Veliceasa D. Ivanovic M. Hoepfner F. T. S. Thumbikat P. Volpert O. V. Smith N. D. (2007). Transient potential receptor channel 4 controls thrombospondin-1 secretion and angiogenesis in renal cell carcinoma. FEBS J. 274, 6365–6377. 10.1111/j.1742-4658.2007.06159.x 18021253

[B41] Vonderheide R. H. (2020). CD40 agonist antibodies in cancer immunotherapy. Annu. Rev. Med. 71, 47–58. 10.1146/annurev-med-062518-045435 31412220

[B42] Wan R. Peng W. Xia Q. Zhou H. Mao X. (2021). Ferroptosis‐related gene signature predicts prognosis and immunotherapy in glioma. CNS Neurosci. Ther. 27, 973–986. 10.1111/cns.13654 33969928 PMC8265949

[B43] Wilkerson M. D. Hayes D. N. (2010). ConsensusClusterPlus: a class discovery tool with confidence assessments and item tracking. Bioinformatics 26, 1572–1573. 10.1093/bioinformatics/btq170 20427518 PMC2881355

[B44] Wm L. Cj R. (2019). The cancer Genome Atlas of renal cell carcinoma: findings and clinical implications. Nat. Rev. Urol. 16, 539–552. 10.1038/s41585-019-0211-5 31278395

[B45] Wu Y. Miyamoto T. Li K. Nakagomi H. Sawada N. Kira S. (2011). Decreased expression of the epithelial Ca2+ channel TRPV5 and TRPV6 in human renal cell carcinoma associated with vitamin D receptor. J. Urol. 186, 2419–2425. 10.1016/j.juro.2011.07.086 22019165

[B46] Wu L. Lian W. Zhao L. (2021). Calcium signaling in cancer progression and therapy. FEBS J. 288, 6187–6205. 10.1111/febs.16133 34288422

[B47] Xu Z. Xiang L. Wang R. Xiong Y. Zhou H. Gu H. (2021). Bioinformatic analysis of immune significance of RYR2 mutation in breast cancer. Biomed. Res. Int. 2021, 8072796. 10.1155/2021/8072796 34888385 PMC8651385

[B48] Xu H. Zheng X. Zhang S. Yi X. Zhang T. Wei Q. (2021). Tumor antigens and immune subtypes guided mRNA vaccine development for kidney renal clear cell carcinoma. Mol. Cancer 20, 159. 10.1186/s12943-021-01465-w 34872567 PMC8645676

[B49] Yin X. Wang Z. Wang J. Xu Y. Kong W. Zhang J. (2021). Development of a novel gene signature to predict prognosis and response to PD-1 blockade in clear cell renal cell carcinoma. Oncoimmunology 10, 1933332. 10.1080/2162402X.2021.1933332 34262797 PMC8253123

[B50] Zhang P. Xu J. Zhang H. Liu X.-Y. (2021). Identification of TRPV4 as a novel target in invasiveness of colorectal cancer. BMC Cancer 21, 1264. 10.1186/s12885-021-08970-7 34814869 PMC8611894

[B51] Zhang H.-C. Deng S. H. Pi Y. N. Guo J. N. Xi H. Shi X. (2022). Identification and validation in a novel quantification system of ferroptosis patterns for the prediction of prognosis and immunotherapy response in left- and right-sided colon cancer. Front. Immunol. 13, 855849. 10.3389/fimmu.2022.855849 35444656 PMC9014300

[B52] Zhao Z. Zhang M. Duan X. Chen Y. Li E. Luo L. (2018). TRPM7 regulates AKT/FOXO1-Dependent tumor growth and is an independent prognostic indicator in renal cell carcinoma. Mol. Cancer Res. 16, 1013–1023. 10.1158/1541-7786.MCR-17-0767 29545479

[B53] Zhong T. Pan X. Wang J. Yang B. Ding L. (2019). The regulatory roles of calcium channels in tumors. Biochem. Pharmacol. 169, 113603. 10.1016/j.bcp.2019.08.005 31415738

