# Supplementary figures and images for: Prognostic and immunological significance of calcium-related gene signatures in renal clear cell carcinoma
Source: Front Pharmacol. 2022 Dec 14;13:1055841. doi: 10.3389/fphar.2022.1055841 (PMC9795407; doi:10.3389/fphar.2022.1055841)

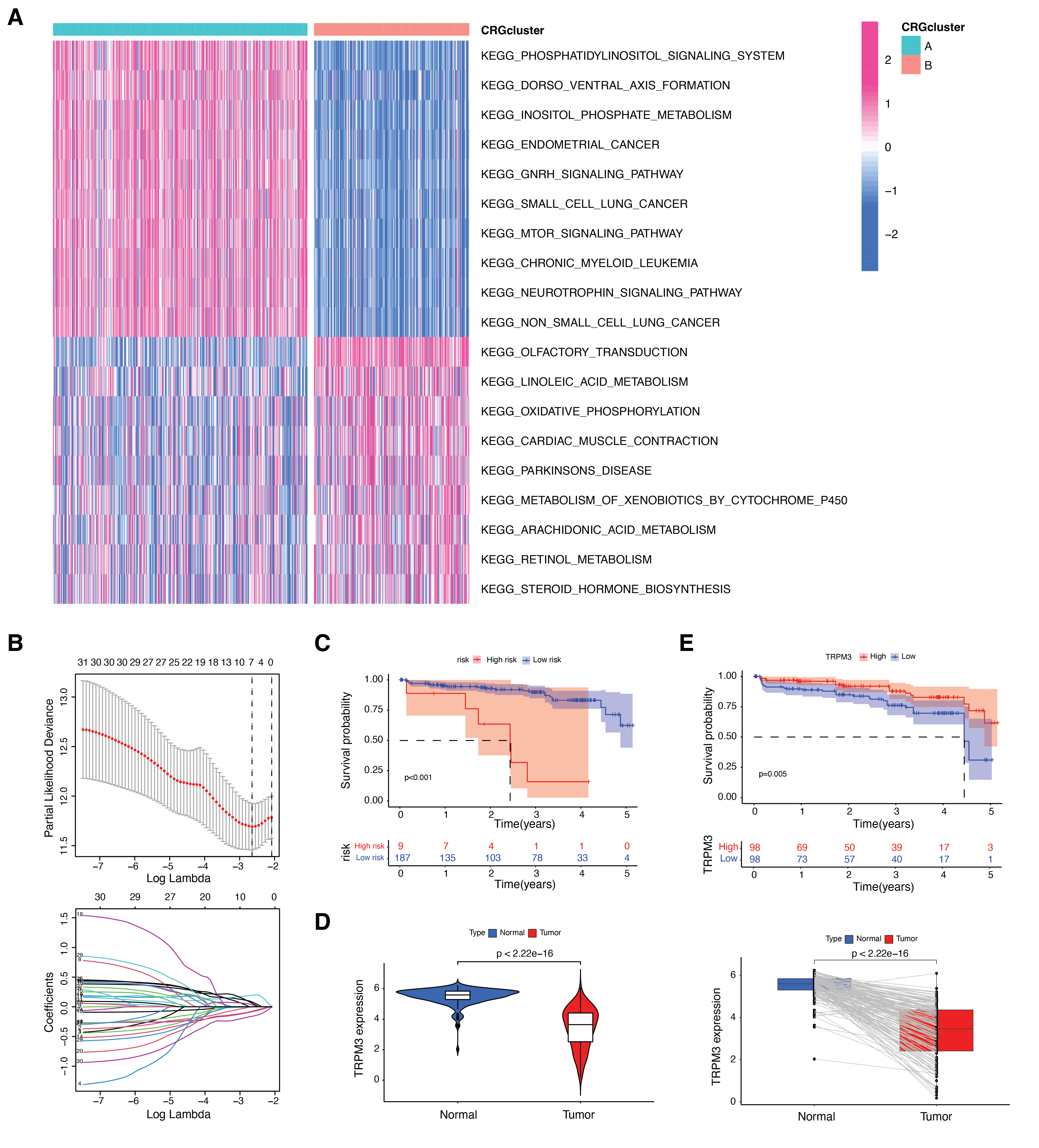

Supplement: Supplementary file 3 [file Image1.tif]
